# Guanosine Mechanisms of Action: Toward Molecular Targets

**DOI:** 10.3389/fphar.2021.653146

**Published:** 2021-03-31

**Authors:** Caio M. Massari, Mariachiara Zuccarini, Patrizia Di Iorio, Carla I. Tasca

**Affiliations:** ^1^ Laboratório De Neuroquímica-4, Departamento De Bioquímica, Centro De Ciências Biológicas, Universidade Federal De Santa Catarina, Florianópolis, Brazil; ^2^ Department of Biomedical Sciences, University G. D’Annunzio of Chieti-Pescara, Chieti, Italy

**Keywords:** guanosine, adenosine receptors, receptors oligomerization, A1R/A2AR heteromers, neuroprotection

## Introduction

Over the years, increasing data concerning the extracellular effect of guanine-based purines have been published. This class of molecules that embraces the well-known nucleotides GTP, GDP and GMP, the nucleoside guanosine (GUO) and the nucleobase guanine are necessary for the maintenance of important intracellular processes, such as nucleic acid structure, energetic metabolism, and signal transduction via G-proteins (Hepler and Gilman 1992). Besides that, guanine-based purines extracellular effects are also noteworthy, mainly through its nucleoside GUO.

In the central nervous system (CNS), GUO acts as a neuromodulator mediating several cellular processes, including cell growth, differentiation and survival (Lanznaster, et al., 2016; Schmidt, Lara, and Souza 2007). Also, GUO exerts protective effects in several models of neurotoxicity or neurological disorders (both *in vitro* and *in vivo*). GUO presents anxiolytic (Bettio et al., 2014), antidepressant-like (Bettio et al., 2012), antinociceptive (Schmidt et al., 2010), and anticonvulsant effects (Lara et al., 2001) in rodents. GUO treatment exerts neuroprotection on Alzheimer’s and Parkinson’s disease *in vivo* models, ameliorating behavior, cognitive and motor function (Su et al., 2009; Massari et al., 2017; Lanznaster, et al., 2016; Marques et al., 2019; da Silva et al., 2020). *In vitro* studies showed that GUO modulates glutamate uptake, decreases the production of reactive oxygen species (ROS), improves mitochondrial function and presents anti-inflammatory properties (Dal-Cim et al., 2012; Marques, et al., 2019; Frizzo et al., 2003; Dal-Cim et al., 2019). Regarding trophic effects, GUO increases the number of neurons in culture (Decker et al., 2019) and recently it was shown that GUO promotes neural stem cell proliferation and neuronal differentiation *in vitro*. Additionally, GUO *in vivo* treatment increases the number of dividing cells and also increases neurogenesis in the hippocampal dentate gyrus (Piermartiri et al., 2020).

### Guanosine Interaction Sites

The intracellular signaling pathways related to GUO effects were the first targets to be identified. It was already shown that GUO effect on cell proliferation is dependent on cyclic AMP (cAMP) level increase (Gysbers and Rathbone 1996; Su et al., 2009). Also, some protective effects are directly related to Phosphoinositide 3-kinase/Protein kinase B (PI3K/Akt) pathway (Dal-Cim et al., 2013; Dal-Cim et al., 2012; Molz et al., 2011; D'Alimonte et al., 2007; Giuliani et al., 2015) and the mitogen-activated protein kinase/extracellular signal-regulated kinase (MAPK/ERK) pathway (Di Iorio et al., 2004; Dal-Cim et al., 2011). Recently, the involvement of protein kinase C (PKC) was also identified, as GUO effect of increasing the glial glutamate transporter-1 (Glt-1) membrane expression after an oxygen/glucose deprivation (OGD) protocol in astrocytes is abolished by PKC or MAPK/ERK inhibition (Dal-Cim et al., 2019).

Since GUO effects evoke intracellular signaling pathways, the need for a membrane receptor target is claimed. The effects of GUO are not altered by nucleosides transporter blockers (Oleskovicz et al., 2008; Giuliani et al., 2015; Decker et al., 2019), indicating that its effects are mediated through interaction with some molecular target at the cellular membrane not yet identified. Some studies pointed to a putative selective GUO receptor in rat brain membranes through [^3^H]GUO binding analysis (Traversa et al., 2002; Traversa et al., 2003). Additionally, a study from Volpini and collaborators suggested GUO interaction with a G-protein coupled receptor (GPCR) (Volpini et al., 2011). In this line of evidence, the pharmacological blockade of GUO neuroprotective effect against ischemia-like *in vitro* protocol with Pertussis toxin also indicates a GPCR interaction (Dal-Cim et al., 2013). While the extracellular effects of purinergic adenine-based nucleotides and nucleoside are better characterized and their respective P2 and P1 receptors are recognized (Palmer and Stiles 1995), guanine-based purines are still orphan molecules.

Apart from the purinergic system, it was shown that GUO effects may depend on a potassium channel interaction. GUO effects of increasing cellular viability in hippocampal slices subjected to OGD and in SH-SY5Y neuroblastoma cells subjected to mitochondrial damage are blocked by large (big) conductance calcium-activated potassium channels (BK) inhibitors (Oleskovicz et al., 2008; Dal-Cim et al., 2012; Dal-Cim et al., 2013). A couple of studies also suggested the relation of GUO effects with GPR23 (Di Liberto et al., 2012) or CD40 receptors (D'Alimonte et al., 2007). Although there is a suggestion for GUO receptors (a selective one, or other putative receptors), a great number of results support that GUO effects are mediated by adenosine receptors (Lanznaster, et al., 2016).

### Guanosine Interaction With Adenosine Receptors

Adenosine plays a pivotal role as a neuromodulator and presents neurotrophic effects acting through its P1 receptors, which are composed of four different GPCRs (A_1_R, A_2A_R, A_2B_R, and A_3_R). A_1_R and A_3_R are typically coupled to Gi proteins and thus inhibit adenylyl cyclase activity, whereas A_2A_R and A_2B_R are coupled to Gs proteins and increase the production of cAMP (Zimmermann, 2011). P1 receptors are expressed in neurons, astrocytes, oligodendrocytes and microglia and their stimulation activates multiple functions, such as synaptic plasticity and presynaptic neuromodulation (Daré et al., 2007; Burnstock, Fredholm, and Verkhratsky 2011; Burnstock and Ulrich 2011). In addition, A_1_R and A_2A_R are the main responsible for adenosine actions on the CNS, while A_1_R is well expressed in the whole brain, A_2A_R is enriched in some particular areas such as the striatum, hippocampus, *raphe nuclei* and *locus coeruleus* (Palmer and Stiles 1995). And, to our knowledge, besides one study showing that a preferential A_2B_R antagonist partially decreased the mitogenic activity of GUO in astrocytes (Ciccarelli et al., 2000), only A_1_R and A_2A_R have been related to GUO effects.

Some results directly imply GUO effects with the A_1_R. Most of GUO known effects are abolished by previous incubation or treatment with the selective A_1_R antagonist DPCPX. *In vitro* protocols of brain ischemia in hippocampus slices and cortical astrocyte cultures demonstrated that DPCPX abolished the protective effects of GUO in ROS production, glutamate uptake, and cell viability (Dal-Cim et al., 2013; Dal-Cim et al., 2019). Similarly, DPCPX prevented GUO protective effect against mitochondrial oxidative stress in human neuroblastoma SH-SY5Y cells (Dal-Cim et al., 2012). Also, it was shown that in primary culture of both neurons and astrocytes, GUO increases global Small Ubiquitin-like MOdifier (SUMO)2/3-ylation at neuroprotective concentrations, an effect abolished by DPCPX preincubation (Zanella et al., 2020). *In vivo* protocols also display the same pattern related to A_1_R. In a reserpine-treated mice protocol, where animals develop a parkinsonian tremor and striatal damage, GUO reverses the motor impairment and decreases ROS level in the striatum, but GUO efficacy is lost when animals are pretreated with DPCPX (Massari et al., 2020). In a traumatic brain injury model in rats, it was seen that mitochondrial dysfunction in the cerebral cortex is reversed by GUO treatment. However, this effect is no longer seen if the animals are pretreated with DPCPX (Gerbatin et al., 2019). In the same way, DPCPX reversed the anxiolytic-like effect induced by GUO, as well as the GUO capacity of decreasing the synaptosomal K^+^-stimulated glutamate release (Almeida et al., 2017). It is important to mention that DPCPX is also considered to be an inverse agonist of A_1_R (Weyler et al., 2006). Additionally, reports are now revealing some molecules that display a biased agonism (a ligand-dependent differential intracellular signaling) on A_1_R, an issue that still needs additional studies (Vecchio et al., 2018). Taken together, these data could suggest that GUO effects are mediated by A_1_R activation. However, in heterologous transfection of A_1_R in HEK293 cells, GUO does not induce calcium mobilization as observed with an A_1_R agonist (R-PIA) treatment (as a positive control). Moreover, GUO has no effect upon R-PIA-inducing calcium mobilization through A_1_R (Lanznaster, et al., 2019).

Data regarding GUO dependence on the A_2A_R signaling are conflicting. While most data show that antagonism of A_2A_R has no impact on GUO promoting effects (Almeida et al., 2016; Almeida et al., 2017; Gerbatin et al., 2019; Massari et al., 2020; Zanella et al., 2020) some reports are showing otherwise (Dal-Cim et al., 2012; Decker et al., 2019). Surprisingly, the pharmacological use of the A_2A_R agonist CGS21680 shows a clear counteraction of GUO-mediating effects. Like the pretreatment with an A_1_R antagonist, A_2A_R agonist also abolishes the protective effects of GUO over ROS production, glutamate uptake, and cell viability on those *in vitro* protocols of brain ischemia (Dal-Cim et al., 2013; Dal-Cim et al., 2019). Recently, it was seen that in mice that do not express A_2A_R (i.e. A_2A_R Knock-out mice, A2AR^−/-^) the preventive GUO effect on ROS production and on cell viability is impaired (Lanznaster et al., 2019). Important to notice, GUO *per se* does not induce cAMP increase in HEK293 cells transfected with A_2A_R, neither interfere with cAMP level increase induced by the A_2A_R agonist CGS 26180 (Lanznaster et al., 2019).

The dubious effect of GUO on adenosine receptors can also be interpreted through the oligomeric interaction of these receptors.

### Adenosine Receptors Forming-Oligomers

The understanding of GPCRs physiology and pharmacology has changed in the last 2 decades. This is due to the growing evidence that they can form homomers (homo-oligomerization, from the same GPCRs) and heteromers (hetero-oligomerization of different GPCRs). This oligomerization induces changes in biochemical properties of GPCRs. It is well established that adenosine receptors can form oligomers among themselves and with receptors for other neurotransmitters, such as dopamine receptors (Ginés et al., 2000; Fuxe et al., 2005; Ciruela et al., 2006; Navarro, et al., 2018; Borroto-Escuela et al., 2018; Ferré and Ciruela 2019; Cortés et al., 2019). It is known that A_1_ and A_2A_ receptors form functional oligomers with each other and that the A_1_R-A_2A_R heteromer plays an important role in modulating the control of cortico-striatal function (Ciruela et al., 2006). This control takes place through the activation of the presynaptic A_1_R or A_2A_R, which depends on the concentration of adenosine, as a low concentration would activate A_1_R while a high concentration would activate A_2A_R, resulting in a lesser or greater release of glutamate, respectively (Ciruela et al., 2006). Moreover, the A_1_R-A_2A_R heteromer seems to have a role in glutamate clearance by modulating the expression of the excitatory amino acid transporter 2 (EAAT2) in astrocytes (Hou et al., 2020). Also, adenosine interaction with A_1_R-A_2A_R heteromer in astrocytes has been shown to control extracellular gamma-aminobutyric acid (GABA) uptake via modulation of GABA transporters (Cristóvão-Ferreira et al., 2013). In this way, it is proposed that A_1_R-A_2A_R heteromer works as an adenosine concentration-sensing device that implies a cross-communication between Gi and Gs proteins guided by the C-terminal tail of the A_2A_R (Navarro, Cordomí, Brugarolas, et al., 2018).

The structure of A_1_R and A_2A_R heterodimerization was recently proposed through *in silico* molecular modeling (Navarro et al., 2016; Navarro, Cordomí, Brugarolas, et al., 2018). A_1_R-A_2A_R heteromer may be organized as a tetramer structure composed of two homodimers of A_1_R and two homodimers of A_2A_R. The homodimerization of A_1_R and A_2A_R occurs through the transmembrane (TM) 4/5 interface while the heterodimerization takes place through the TM 5/6 interface of these receptors (Navarro, Cordomí, Brugarolas, et al., 2018).

**FIGURE 1:**
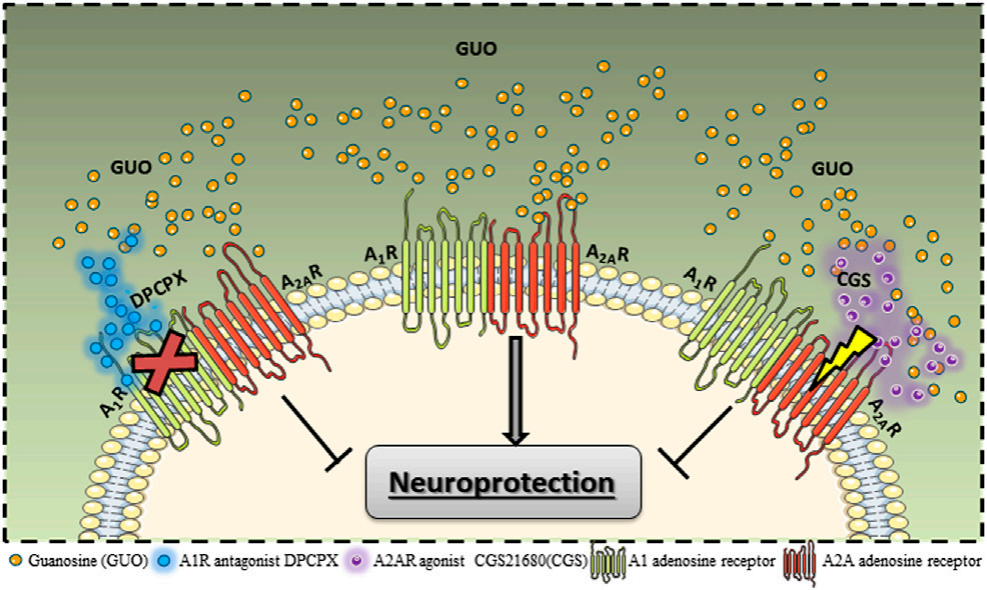
Guanosine (GUO) interaction with adenosine A_1_R-A_2A_R heteromer. Studies with heterologous adenosine receptors transfection showed GUO effects on ligand binding to receptors and intracellular signaling activation require both A_1_R and A_2A_R co-expression. Studies evaluating the neuroprotection promoted by GUO showed the protective is effect is abolished by A_2A_R agonist (CGS21680) and A_1_R antagonist (DPCPX). This pharmacological modulation also points to an interaction with the A_1_R-A_2A_R heteromer.

In this line, using an *in vitro* approach with transfected HEK293 cells, we recently showed that GUO-induced effects require both A_1_R and A_2A_R co-expression. GUO was able to decrease A_2A_R binding affinity and cAMP response evoked by a selective A_2A_R ligand but only in cells expressing both A_1_R and A_2A_R. Also, GUO had no effect on A_1_R signaling in the presence or absence of A_2A_R co-expression (Lanznaster et al., 2019). Thereby, we interpret that GUO interacts with the adenosine receptors as a heteromeric entity, thus the most adjusted hypothesis is that GUO could be acting as a negative modulator of A_2A_R, but only in the presence of A_1_R. It is feasible to speculate that the physical interaction between A_1_R and A_2A_R could lead to an increase of A_2A_R affinity for GUO. CGS21680 could be directly interfering in the GUO signaling on A_2A_R, whereas DPCPX interacting with A_1_R may be responsible for allosteric modulation of GUO A_2A_R affinity upon the A_1_R-A_2A_R heteromers (Figure 1). Indeed, GUO modulation over other adenosine-forming heteromers could not be discarded and might be different among brain structures, once that it may depend on differential receptors expression. Intriguingly, the GUO protective effect is lost in hippocampal but not in striatal slices from A2AR^−/-^ mice (Lanznaster et al., 2019; Massari et al., 2020). Therefore, more studies regarding GUO interactions with adenosine oligomers are necessary.

## Conclusions and Perspectives

The increasing evidence supporting GUO protective action and trophic effects in the CNS are undeniable. This nucleoside is still considered an orphan neuromodulator, although its importance as an integrative molecule between purinergic and glutamatergic transmission. Some evidences suggest a selective GUO interaction site, whereas several studies show a dependence of GUO effects on adenosine receptors interaction. Considering the new paradigms related to adenosine receptors pharmacology (as allosterism, bias agonism and oligomeric interactions), there is a multitude of new interaction sites to be explored. These new insights of GUO interaction within GPCR heteromerization and the understanding of GUO effects on adenosine A_1_R-A_2A_R heteromers could open a new window in therapeutic approaches toward purinergic signaling.

## **Abbreviations**

A1R, adenosine A1 receptors; A2AR, adenosine A2A receptors; cAMP, cyclic AMP; CGS21680, 4-(2-[7-Amino-2-(2-furyl)[1,2,4] triazolo[2,3-a][1,3,5]triazin-5-ylamino]ethyl) phenol; CNS, central nervous system; DPCPX, 1,3-dipropyl-8-cyclopentylxanthine; GPCR, G-protein coupled receptor; GUO, Guanosine; OGD, oxygen/glucose deprivation; ROS, reactive oxygen species; R-PIA, R(−)-N6-(2-phenylisopropyl)adenosine; SUMO, Small Ubiquitin-like Modifier.
